# Notes and News

**Published:** 1901-10-26

**Authors:** 


### Notes and News.

We learn that the King, who recently consented to become patron of the new Dental Hospital of London, Leicester Square, has now commanded that in future the institution shall be known as the *Royal* Dental Hospital of London. The new hospital is now finished and in full working order.

It is with great pleasure that we note the signs which are continually cropping up of that fusion and consolidation of the English-speaking race which seems likely to be a distinguishing feature of the opening century. Hence it is not entirely without emotion that we note on the front page of the New York *Medical Record*, among the titles of the leading articles one which stands as follows: "The Health of the King." *The* King. Admirable!

We are officially informed that Surgeon-General W. Taylor, M.D., C.B., K.H.P., has been appointed Director-General of the Army Medical Service, in succession to Surgeon-General Jameson. The new Director-General, who has been at the head of the Indian branch of the Army Medical Service for some years, is 58 years of age, and has a high reputation for administrative ability.

Major Walter Reed has submitted an interesting report to the United States War department on his conclusions as to the infection of yellow fever, resulting from his experience at Santiago. Since 1899, when there was an epidemic with a mortality of 20 per cent., there has been no yellow fever in Santiago. The United States Government instituted a rigorous disinfection which has proved most effectual. Major Walter Reed unhesitatingly states that the mosquito is responsible for the spread of the fever.

Sir Joseph Dimsdale, M.P., Lord Mayor Elect, has promised to preside at a Mansion House Meeting, to be held early in January next, in furtherance of the appeal of Guy's Hospital for a renewal of public support. A sum of £180,000 is required to meet the cost of many works of renovation and extension, rendered necessary by the age of the hospital and the constantly-increasing demands upon its ministrations. The appeal also asks that the income of the hospital derived from voluntary sources may be increased to £25,000 per annum.

A new fever hospital has been opened at Forfar for the Dundee and Forfar district. The accommodation of 30 beds has been provided for £9,000. The architect is Mr. Cappon, F.R.I.B.A.

The Secretary of State for India has sanctioned an increase of 26 officers to the India Medical Service. It has been somewhat optimistically stated that this will get over the difficulty respecting leave.

That severely criticised institution called the Consultative Institute at Birmingham has died a natural death. The door-plate is gone, the effects have been sold, and we imagine that the promoters of the ill-advised scheme will be glad when the memory of it has passed away.

The Liverpool Sanatorium for Consumption in Delamere Forest has been opened by Lady Derby. The institution is largely indebted to Lady Willox and Mr. W. P. Hartley, who each offered £7,500 towards building the Sanatorium. The main building is practically divided into three blocks, with bungalows, provision being made for 32 patients.

The foundation-stone of a new wing at the Royal Hospital for Incurables has been laid by Mrs. Restell, widow of Mr. M. Restell, who bequeathed £6,000 for the purpose, and after whose name the new wing will be called. Sixteen new beds will be added to the accommodation of the institution, which is always overwhelmed with applications for admission.

Some months ago Mr. Alfred de Rothschild made special appeals on behalf of St. Mary's Hospital, asking that the public should subscribe £25,000 to obtain £25,000 offered on this condition by the trustees of the Zunz bequest. Through his exertions and the great generosity of many others the required sum has been collected, and thus St. Mary's Hospital is the fortunate possessor of £50,000.

Candidates for the Fiske Fund Prize may now send in their essays for 1902. The subject is "Serum-therapy in the Light of the most recent Investigations." The value of the prize is £50. The essays must bear a motto, and be accompanied by an envelope on the outside of which is the motto, and inside the name and address of the writer. The secretary is Dr. Halsey de Wolf, 24 Benefit Street, Providence, Rhode Island, U.S.A.

The prevalence of small-pox in London has called into existence a new league to further vaccination. Its objects are to spread a wider knowledge of the benefits derived from vaccination and a better understanding among the general public of the advantages arising from preventative medicine and practical sanitation. The league already counts among its supporters a number of influential laymen interested in the subject, and numerous eminent medical men, including Mr. Jonathan Hutchinson, F.R.S., member of the recent Royal Commission on Vaccination, Sir Alfred Garrod, F.R.S., Physician extraordinary to her late Majesty Queen Victoria, and Professor Charles Stewart, F.R.S., of the Royal College of Surgeons. The temporary offices of the league are at 110 Strand, London, W.C.

